# Revitalizing the Gut Microbiome in Chronic Kidney Disease: A Comprehensive Exploration of the Therapeutic Potential of Physical Activity

**DOI:** 10.3390/toxins16060242

**Published:** 2024-05-26

**Authors:** Marieke Vandecruys, Stefan De Smet, Jasmine De Beir, Marie Renier, Sofie Leunis, Hanne Van Criekinge, Griet Glorieux, Jeroen Raes, Karsten Vanden Wyngaert, Evi Nagler, Patrick Calders, Diethard Monbaliu, Véronique Cornelissen, Pieter Evenepoel, Amaryllis H. Van Craenenbroeck

**Affiliations:** 1 Nephrology and Renal Transplantation Research Group, Department of Microbiology, Immunology and Transplantation, KU Leuven, 3000 Leuven, Belgium; marieke.vandecruys@kuleuven.be (M.V.); or pieter.evenepoel@uzleuven.be (P.E.); 2 Exercise Physiology Research Group, Department of Movement Sciences, KU Leuven, 3000 Leuven, Belgium; stefan.desmet@kuleuven.be; 3 Department of Rehabilitation Sciences, Ghent University, 9000 Ghent, Belgium; jasmine.debeir@ugent.be (J.D.B.); patrick.calders@ugent.be (P.C.); 4 Group Rehabilitation for Internal Disorders, Department of Rehabilitation Sciences, KU Leuven, 3000 Leuven, Belgium; marie.renier@kuleuven.be (M.R.); veronique.cornelissen@kuleuven.be (V.C.); 5 Department of Microbiology, Immunology and Transplantation, Abdominal Transplantation, KU Leuven, 3000 Leuven, Belgium; sofie.leunis@kuleuven.be (S.L.); hanne.vancriekinge@kuleuven.be (H.V.C.); diethard.monbaliu@kuleuven.be (D.M.); 6 Department of Internal Medicine and Pediatrics, Nephrology Section, Ghent University Hospital, 9000 Ghent, Belgium; griet.glorieux@ugent.be (G.G.); karsten.vandenwyngaert@uzgent.be (K.V.W.); evi.nagler@uzgent.be (E.N.); 7 Department of Microbiology and Immunology, Rega Institute for Medical Research, 3000 Leuven, Belgium; jeroen.raes@kuleuven.be; 8 VIB-KU Leuven Center for Microbiology, 3000 Leuven, Belgium; 9 Transplantoux Foundation, 3000 Leuven, Belgium; 10 Department of Nephrology, University Hospitals Leuven, 3000 Leuven, Belgium

**Keywords:** chronic kidney disease, gut microbiome, physical activity, exercise

## Abstract

Both physical inactivity and disruptions in the gut microbiome appear to be prevalent in patients with chronic kidney disease (CKD). Engaging in physical activity could present a novel nonpharmacological strategy for enhancing the gut microbiome and mitigating the adverse effects associated with microbial dysbiosis in individuals with CKD. This narrative review explores the underlying mechanisms through which physical activity may favorably modulate microbial health, either through direct impact on the gut or through interorgan crosstalk. Also, the development of microbial dysbiosis and its interplay with physical inactivity in patients with CKD are discussed. Mechanisms and interventions through which physical activity may restore gut homeostasis in individuals with CKD are explored.

## 1. A Healthy Gut Microbiome

### 1.1. Characteristics of a Healthy Gut Microbiome

The gut microbiome plays a pivotal role in maintaining health as well as in the pathogenesis of disease [1,2,3]. The gut microbiota specifically refers to the living microorganisms that reside in the gut, while the gut microbiome encompasses a broader spectrum. It includes not only the microbiota but also their collective genome, comprising bacteria, archaea, viruses, and fungi, along with their theater of activity. The latter refers to microbial structural elements, metabolites, nucleic acids (including mobile genetic elements like viruses, phages, and residual DNA), and the surrounding environmental conditions [3,4,5]. The gut microbiota can be taxonomically classified by species, genus, family, order, class, and phylum [5,6]. The relative abundance of operational taxonomic units, also called gut microbiota composition, is host-specific [2]. However, culture-based studies suggest that the majority of species of gut microorganisms are similar in healthy adults, referred to as the core microbiota [7,8]. The core microbiota of healthy individuals is characterized by high gut microbiome diversity, which refers to the amount (richness) and distribution (evenness) of different species in the gut microbiome community [9,10]. A healthy core microbiota is characterized by low levels of pro-inflammatory bacteria and high levels of short-chain fatty acid (SCFA)-producing bacteria [11]. Bacterial-derived SCFAs such as acetate, propionate, and butyrate are products of dietary fiber fermentation [12], support the expression of tight junction proteins in colon epithelia, and act as an energy source for colonocytes, thereby aiding in the preservation of the intestinal barrier [13]. Through preserving intestinal barrier cohesion, SCFAs contribute to the inhibition of an inflammatory response caused by the release of endotoxins such as lipopolysaccharide (LPS) in the circulation [12]. An adequate balance of bacteria in the digestive tract ensures that the microbiota function harmoniously and in a symbiotic relationship with the host [14,15]. On the other hand, gut dysbiosis, defined as a disturbance in gut microbiota equilibrium, arises from imbalances in the microbiota itself, shifts in their functional capacity and metabolic activity, or alternations in their local distribution [16,17]. A healthy, well-functioning gut microbiome not only contributes to the digestion of food and maintaining intestinal barrier integrity but also serves as a potent modulator of bile salt deconjugation, vitamin production, immunity, and metabolic health [4,18,19]. The gut microbiome of an adult human seems to be shaped by both host-related and environmental factors, such as genetics, antibiotic therapy, diet, physical activity level, and other lifestyle habits [4,20].

### 1.2. An Active Lifestyle, a Healthy Gut?

Physical activity (PA) and exercise modify the gut microbiome independently of diet [21]. PA encompasses a broad spectrum of activities, as further discussed in Table 1. In rodent models, exercise modifies the composition and diversity of microorganisms living in the gut towards more health-beneficial taxa [6,21,22,23]. Exercise training has been shown to be effective in changing the gut microbiota’s metabolic activity in controlled animal trials (reviewed elsewhere [21]). Matsumoto et al. were the first to report an increase in butyrate-producing bacteria together with an increase in butyrate production in the cecum following 5 weeks of exercise training in rats [24]. Various studies corroborate this finding, demonstrating that regular exercise increases the relative abundance of SCFA-producing taxa in rodents [21]. Studies conducted in obese and healthy mice demonstrated that voluntary wheel running increased intestinal barrier integrity and diminished intestinal inflammation [22,25]. In humans, individuals who engage in PA portray a distinct gut microbiota signature compared to their physically inactive counterparts [6]. Observational data showed that athletes possess a different gut microbiota composition and increased diversity, with a greater abundance of health-associated taxa such as lactate utilizers and SCFA producers, which in turn leads to greater fecal SCFA concentrations [18,26,27]. Athletes show a higher density of *Akkermansia muciniphila*, which are mucin-degrading bacteria that improve intestinal barrier integrity by upregulating tight junction proteins and boosting anti-inflammatory T regulatory cells [18,28,29]. In that respect, more and more research advocates that intestinal barrier integrity improves with regular exercise [29,30]. In a cross-sectional design, trained cyclists displayed three-fold lower plasma LPS at rest relative to healthy sedentary individuals [31]. Also, exercise intervention studies in healthy humans have shown exercise to modify gut microbiota composition, with a decline in the *Firmicutes/Bacteroidetes* ratio and an increase in *Bacteroides* and *Roseburia* genera (a butyrate producer) [32]. Human intervention studies, i.e., moderate- and high-intensity aerobic or high-intensity combined aerobic and strength training, have shown an increase in gut microbiome diversity, with an increased abundance of SCFA-producing genera and corresponding fecal SCFA concentrations in healthy adults [33,34,35]. Regarding intervention studies in the obese population, exercise improved diversity as well as the abundance of specific species, with lower numbers of *Proteobacteria phylum* and higher numbers of *Bacteroides*, *Bifidobacteriaceceae*, and *Akkermansia* genera [36].

Regretfully, the impact of the various PA dimensions on the gut microbiome has only been briefly examined in clinical research, with the majority of alterations occurring during aerobic exercise [37]. However, a small influence is seen by resistance training, which may compound the effects of aerobic exercise on the gut microbiome [37]. The World Health Organization’s minimal dose of PA, which is advised for persons aged 18 to 64, seems to cause some changes in the composition of the gut microbiome, but not significantly in terms of richness and diversity [37,38]. Moderate to high-intensity exercise interventions for more than 30 min, 3 or more times per week, and for more than 8 weeks result in the most consistent changes in the human gut microbiota [28,37]. However, excessive or prolonged exercise may negatively impact the gut microbiome by increasing some commensal bacteria, increasing potential harmful bacteria, and decreasing gut microbial diversity [19,39].

**Table 1:** Concepts of physical activity.

Physical activity (PA) [40]	Any skeletal movement that results in energy expenditure, including all bodily movements produced during recreational time, transport, or occupational activities
Exercise [40]	A subset of PA that is planned, structured, and repetitive and has the objective to improve or maintain physical fitness
Exercise intensity [41]	PA can be classified based on the amount of energy expenditure or metabolic equivalent of the task (MET), into low intensity PA (<3 METs), moderate intensity PA (3–6 METs) or high intensity PA (>6 METs)
Aerobic exercise [42,43]	Involve continuous rhythmic movements that elevate breathing frequency and heart rate, also referred to as endurance exercise
Strength exercises [44]	Aim to enhance muscle strength and mass, typically through resistance training where muscles work against a force
Balance exercises [43]	Help improve stability and coordination by challenging the body’s ability to maintain equilibrium
Flexibility exercises [43]	Focus on improving range of motion in muscles and joints

### 1.3. Mechanisms via Which PA Modifies the Gut Microbiome

**Figure 1:**
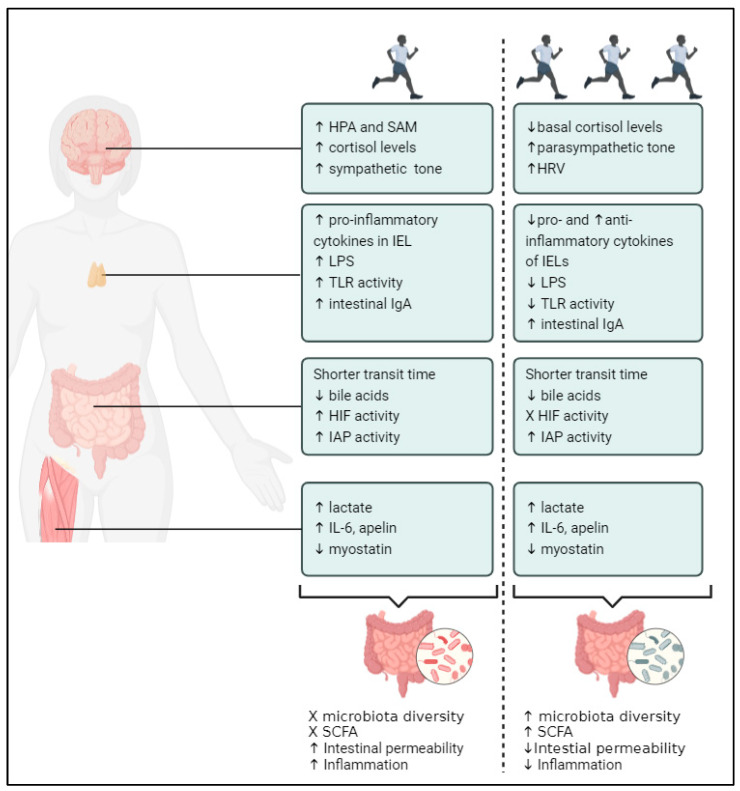
Effects of acute and chronic exercise on the gut microbiome in the healthy population. Acute exercise refers to a single bout or session of PA, while chronic exercise refers to long-term, regular engagement in PA. HPA: hypothalamic–pituitary–adrenal axis; SAM: sympatho-adreno-medullary; HRV: heart rate variability; IEL: intraepithelial lymphocytes; LPS: lipopolysacharides; TLR: toll-like receptors; IgA: immunoglobulin A; HIF: hypoxia inducible factor; IAP: intestinal alkaline phosphatase; IL-6: interleukin 6; SCFA: short-chain fatty acids.

This section discusses the underlying pathways via which PA might influence the gut microbiome in both animals and healthy individuals across four different levels, i.e.,: the gut, the immune system, the nervous system, and the muscle (as visualized in Figure 1). 

#### 1.3.1. PA and Gastrointestinal Fitness

PA may alter the gut microbiome by influencing intestinal transit time, a critical factor in establishing the makeup and activity of the gut microbiota [45,46]. Transit duration has been related to gut microbial composition, diversity, and metabolic activity in both animal and population-wide as well as small-scale studies [45,47]. Asnicar et al. found that several microbial species, including *Akkermansia muciniphila*, *Bacteroides* spp., and *Alistipes* spp., were associated with longer gut transit times in healthy adults [48]. Since variations in transit time have an impact on substrate availability throughout the intestinal lumen, intestinal transit time consequently influences the metabolism of gut microbes [45]. According to Roager et al., greater levels of protein-derived metabolites in the urine of humans with prolonged intestinal transit time suggest a shift in colonic metabolism from carbohydrate fermentation to protein catabolism [49]. Additionally, there is a correlation between shorter intestinal transit times and higher fecal butyrate concentrations in healthy individuals [45,50], as well as other metabolites that may indicate a higher rate of colonic mucosal renewal [49]. A shorter intestinal transit time may lessen the amount of time that pathogens spend in contact with the gastrointestinal mucus layer and the circulatory system [51,52]. Studies in both healthy adults and adults with chronic idiopathic constipation showed that regular PA shortened intestinal transit time [46,53,54,55]. In addition, trained runners who underwent a week of intense training showed reduced intestinal transit times after exercise [56].

Furthermore, longer intestinal transit times in humans are linked to higher distal intestinal pH, indicating a relationship between both [45]. Significant amounts of lactate are released into the gut lumen during high-intensity exercise, which changes the pH of the intestinal lumen [57]. However, due to the fact that lactate is a byproduct of energy metabolism in the muscle, this issue is further discussed below. 

Alternations in the enterohepatic circulation of bile acids are another mechanism by which PA may influence the gut microbiome. The gut microbiota–bile acid axis refers to the complex relationship that exists between the gut microbiota and bile acids in the host [58]. While primary bile acids are produced by hepatocytes and are stored in the gall bladder, secondary bile acids are produced by gut bacterial metabolism [59]. The latter are regular metabolites that are essential for lipid absorption and digestion, as well as the uptake of fat-soluble vitamins and cholesterol [60]. Moreover, bile acids have the ability to successfully control the gut microbiota and preserve intestinal homeostasis [58]. In turn, the gut microbiota has the ability to impact bile acid synthesis and change bile acid receptor signaling [58]. The main bacterial genera involved in bile acid metabolism include *Bacteroides*, *Peptostreptococcus*, *Lactobacillus*, *Bifidobacterium*, *Listeria*, *Eubacterium*, *Clostridium*, and *Fusobacterium* [61]. Interestingly, time spent in recreational PA was shown to be adversely correlated with total fecal bile acid concentrations in a cross-sectional examination of the baseline fecal bile acid levels of 735 people with colorectal adenoma. Fecal bile acid concentrations were 17% lower in participants in the highest quartile of PA than in those in the lowest quartile [62]. Similarly, one observational study showed that the fecal bile acid concentration in distance runners was lower in comparison with healthy controls [63]. With regards to intervention studies, Danese et al. revealed that the serum concentration of total bile acids in amateur runners’ was lower following a half-marathon run [64]. Osuna-Prieto et al. and Morville et al. also found decreased total plasma levels of primary and secondary bile acids in young sedentary adults and young trained males after a bout of endurance or resistance training [65,66]. These findings are of importance due to the fact that bile acid circulation is a major regulator of gut microbial communities [59]. However, the mechanism by which PA alters bile acid concentrations is not yet fully understood [67]. It is commonly known that endurance exercise causes an instantaneous drop in blood cholesterol levels, which has been confirmed in middle-distance runners [64]. Therefore, during endurance exercise, the reduced availability of cholesterol in the blood could serve as a barrier to the liver’s capacity to synthesize primary bile acids efficiently [64].

PA might alter the gut microbiome through gastrointestinal hypoperfusion and the accompanied activation of hypoxia-inducible factor (HIF). Adequate intestinal blood flow is crucial for the proper functioning of the gastrointestinal system, ensuring oxygen and nutrient delivery while removing metabolic by-products [68]. The gastrointestinal tract has a distinct oxygenation profile and undergoes significant fluctuations in blood perfusion [69]. It has been demonstrated that there are steep oxygen gradients from the anaerobic lumen through the epithelium and into the highly vascularized subepithelial mucosa [69]. Two main processes contribute to the gut oxygen environment: (i) oxygenation by the blood stream; and (ii) the oxygen consumption of both host cells and microbial components [70]. The latter mostly consists of anaerobes that reduce ambient oxygen levels, and as a result, the intestine is extremely hypoxic relative to other tissues [71]. More specifically, microbiota-derived metabolites increase oxygen consumption by intestinal epithelial cells, decreasing the amount of oxygen available in the gut and causing hypoxia. Consequently, cellular hypoxic sensors are triggered, which modify the metabolism and functionality of intestinal epithelial cells and mucosa-resident cells [70]. Thus, despite the anaerobic nature of the stomach, gut epithelial cells mainly utilize oxidative metabolism [70]. Therefore, the intestine is highly dependent on the adaptive pathways activated by hypoxia [71]. In order to maintain oxygen homeostasis, HIF facilitates both oxygen delivery and adaptation to oxygen deprivation in numerous cell types, including intestinal epithelial cells [69]. HIF, consisting of an alpha and a beta subunit, plays a crucial role in responding to low oxygen levels by regulating gene expression. Under normal oxygen conditions, the alpha subunit (HIFα) undergoes degradation facilitated by prolyl hydroxylases. However, when oxygen is scarce, prolyl hydroxylase activity is inhibited, leading to the accumulation of HIFα and the initiation of downstream transcription processes [68,69,70]. In an HIF-dependent way, hundreds of genes are both positively and negatively regulated in response to hypoxia [69]. Moreover, intestinal epithelial cell metabolism, intestinal barrier modulation, and gut microbiota segregation in humans and mice are all impacted by hypoxia and HIF responses [70]. Mouse models subjected to intermittent hypoxia, which is proven to cause gastrointestinal hypoperfusion, had altered gut microbial communities. Compared to controls, mice subjected to hypoxia had a greater abundance of *Firmicutes* and a lower abundance of *Bacteroidetes* and *Proteobacteria phyla* [72]. Additionally, severe problems in the integrity of the mucosal intestinal barrier are shown by both cell models and mice models with a knockdown of HIF-1α intestinal epithelial cells [71]. The formation of mucus is an important protective mechanism for the commensal population. Mucus is secreted by specialized intestinal epithelial cells known as goblet cells. Mucins are the main glycoproteins in mucus, and HIF-1α directly controls their transcription [71]. HIF-1α activation regulates gut bacterial composition through the production of antimicrobial peptides and stabilizes intestinal barrier function via upregulation of P-glycoprotein and tight junction proteins [70]. HIF-1 is hydroxylated to create a complex with HIF1α, which then triggers the transcription of mucin-1 proteins by triggering MUC genes found in intraepithelial cells. These genes produce mucus and stabilize the integrity of the intestinal barrier against severe pathogenic attacks [73]. Acute, intense exercise has been demonstrated to decrease splanchnic blood flow in both mice and humans, leading to hypoxia in the abdominal organs [68]. Acute exercise can result in a reduction in intestinal blood flow by over 50%, and within just 10 min of high-intensity exercise, significant gut ischemia may occur [74]. Unfortunately, there is limited research on the activation of HIF responses by acute and chronic exercise. However, one study in rodents indicated that a single bout of moderate exercise could elevate intestinal HIF-1α levels, suggesting that exercise might induce HIF-1α accumulation in the lower gastrointestinal system [68].

A small intestinal brush border enzyme called intestinal alkaline phosphatase (IAP) serves as a barrier for the gut mucosa, preventing the translocation of gut bacteria to mesenteric lymph nodes, regulating bicarbonate secretion, detoxifying LPS, dephosphorylating pro-inflammatory nucleotides, and regulating gut microbes [75,76]. The latter is shown by, among others, Malo et al., who concluded that the feces of IAP-deficient mice had significantly different aerobic and anaerobic microbes in comparison to healthy mice. Oral supplementation of IAP promoted the growth of beneficial bacteria, improved the recovery of the gut microbiota that was destroyed by antibiotic therapy, and prevented the formation of harmful bacteria [76]. Sun et al. demonstrated that in mice with type 2 diabetes, eight weeks of aerobic exercise and aerobic exercise combined with resistance training increased the expression of ileal tight junction-related proteins and enhanced IAP activity [77]. Wojcik-Grzybek et al. demonstrated that applying moderate exercise to spinning wheels for seven weeks, along with IAP supplementation, improved experimental colitis in obese mice. In addition, there were modifications to the intestinal microbiota, a weakening of oxidative stress indicators in the colonic mucosa, and a decrease in pro-inflammatory biomarker levels [78]. However, further research is necessary to investigate the effects of PA on IAP and the gut microbiome in humans, along with the underlying mechanisms.

#### 1.3.2. PA and Fitness of the Immune System

Exercise can alter the immune system, with high-intensity acute exercise causing immunosuppression, while chronic moderate exercise improves immune function [79]. The gut-associated lymphoid tissue (GALT) [79], the biggest lymphoid organ in the human body that houses over 70% of all immune cells, is a crucial component of the immune system [80]. More specifically, the lamina propria, mesenteric lymph nodes, isolated lymphoid follicles, intraepithelial lymphocytes, Peyer patches, and other components of the GALT serve as a line of defense against foreign substances that can breach the luminal mechanical barrier [81]. Interestingly, PA alters the GALT [82] and thereby the gut microbiome by the following mechanisms discussed below: 

Intraepithelial lymphocytes (IELs), found inside the intestinal epithelium, serve as the first line of defense against pathogens due to their close proximity to enterocytes and their immediate contact with antigens in the gut lumen [83,84]. Intestinal IELs have been proposed to be crucial modulators of the intestinal epithelia by generating pro-inflammatory cytokines (IFN-γ and TNF-α), anti-inflammatory cytokines (TGF-β and IL-10), and antimicrobial proteins [85,86]. Hoffman-Goetz et al. showed that 16 weeks of voluntary exercise training significantly elevated the expression of the endogenous antioxidants glutathione peroxidase-1 and catalase in the intestinal IELs of mice [83]. Superoxide dismutase levels in the intestinal IELs of mice were elevated in trained mice compared to the control group, albeit not significantly [83]. As a second finding, voluntary wheel running exercise in mice was found to decrease intestinal IELS of the transcription factor NF-kB and to decrease the expression of the pro-inflammatory cytokine TNF-α. Lastly, compared to untrained controls, the long-term training routine was linked to noticeably higher intestine IEL counts [83]. Packer et al. discovered that in healthy older mice, 4 months of wheel running was linked to lower production of the apoptotic protein caspase-7 and the pro-inflammatory cytokine TNF-α in intestinal IELs [87]. According to another study by Hoffman-Goetz et al., prolonged voluntary running increased the expression of pleiotropic IL-6 and anti-inflammatory IL-10 while decreasing the expression of the pro-inflammatory cytokine TNF-α in intestinal IELs [88]. In conclusion, animal research revealed that PA modifies the expression of IEL genes, resulting in a decrease in pro-inflammatory cytokines and an increase in anti-inflammatory cytokines and antioxidant enzymes, which are important modulators of the intestinal mucosal barrier and play a crucial role in the control of intestinal epithelia [83,87,88].

Immunoglobulin A (IgA) also plays an important role in the control of mucosal immunity through a number of processes, i.e., (i) immune exclusion through interaction with environmental antigens; (ii) anti-inflammation through intestinal antigen sampling to elicit Th2 or regulatory T cell-biased mucosal immune responses; and (iii) commensal homeostasis through improved interfacial communication between the intestinal mucosa and probiotic bacteria [82]. IgA can directly bind to and ‘coat’ commensal bacteria in the gut in addition to providing vital defense against pathogens and toxins [89]. Therefore, intestinal IgA may have an impact on colonization levels; however, the mechanisms behind IgA’s impacts on the microbiota remain unclear [90]. IgA-deficient animals showed decreased overall microbial diversity, changed bacterial composition, increased bacterial translocation, and increased vulnerability to intestinal inflammation [89]. On the other hand, mice with changed IgA repertoires or overactive IgA responses also showed altered gut microbiota, compromised mucosal defense, and intestinal symptoms such as decreased metabolism [89]. IgA is critical in shaping the composition of the gut microbiota in mice [91]. Regular moderate PA, such as swimming, has been demonstrated to enhance intestinal levels of IgA in the duodenum and ileum of adult mice’s small intestine [92,93]. Similarly, in adult and senile mice, intestinal IgA concentrations rose with frequent moderate aerobic exercise, such as running [82]. Furthermore, humans secreted more salivary lysozyme and salivary IgA after an exhaustion trial [94]. IgA might play a role in both acute and chronic exercise and is known to alter gut microbiota composition; however, further research is needed to gain insight into the underlying mechanisms. 

Toll-like receptors (TLRs) are essential for host defense against pathogens, controlling the number of commensal bacteria, and preserving intestinal integrity and homeostasis [95,96,97]. Acute, intense exercise can compromise gut integrity and allow gut microbiota or their bacterial products to enter the bloodstream, which can activate TLRs on immune cells and cause low-grade systemic inflammation [98,99]. In this case, LPS, also referred to as endotoxin, is a recognized indicator of sepsis and a part of the membrane of Gram-negative bacteria [100]. When the intestinal barrier is breached, LPS attaches to TLR and initiates a number of signaling cascades, including the transcription factor NF-kB, which in turn triggers the release of pro-inflammatory cytokines that worsen both systemic and intestinal inflammation [100]. Toll-like receptor 2 (TLR2) and Toll-like receptor 4 (TLR4) are the most important receptors of LPS, and Toll-like receptor 5 (TLR5) is a specific receptor of the bacterial flagellin [101]. The latter is a transmembrane protein that is highly expressed in the intestinal mucosa [102] and is of importance in shaping the gut microbiota [95]. Carvalho et al. showed that TLR5-deficient mice exhibited increased intestinal pro-inflammatory gene expression and colitis with incomplete penetrance [102]. Consequently, Vijay-Kumar et al. documented that TLR5-deficient mice showed significantly different gut microbiota compositions compared to their germ-free wild-type controls [103]. TLR4 is also a shaper of the gut microbiota. Dheer et al. demonstrated that the microbiota of mice is altered in terms of composition and richness in response to increased TLR4 signaling [104]. With regards to exercise, high-intensity acute exercise can increase gut permeability, leading to the penetration of LPS in the circulation and the activation of TLRs [28]. On the contrary, it seems that chronic exercise decreases gut permeability, thereby reducing TLR signaling activation. Li et al. concluded that eight-week aerobic exercise training increased intestinal mucosal barrier function in diabetic rats by inhibiting LPS release and pro-inflammatory cytokine expression [105]. Uchida et al. found that prolonged exercise causes mice’s intestinal cell surface to express TLR5, which in turn increases the production of TNF-α in response to flagellin [99]. In regards to human research, one systematic review [106] reported that Lancaster et al. were the first to show a decline in monocyte (CD14+) TLR expression and function following a single bout of prolonged aerobic exercise [107]. They specifically looked into how 1.5 h of intense cycling activity (~65% VO2 max) in the heat affected the expression and functionality of TLRs in vivo. When compared to samples taken at rest, TLR1, TLR2, and TLR4 expression was significantly lower after two hours of recovery and post-exercise [107]. Moreover, they [106] reported that Flynn et al. discovered that older women who underwent resistance training had considerably lower TLR4 mRNA than older women who were untrained and sedentary [108]. Similarly, individuals who engaged in PA had lower cell-surface TLR4 expression and produced fewer inflammatory cytokines in response to LPS stimulation [109]. In healthy adults, an exercise regimen of combined resistance and aerobic exercise considerably reduced LPS-stimulated inflammatory cytokine production and cell-surface TLR4 expression [110]. However, the hypothesis that exercise alters the gut microbiome through altered TLRs activation is, at present, not proven. 

#### 1.3.3. PA and Fitness of the Nervous System

The gut microbiota–brain axis refers to the bidirectional communication between the enteric nervous system (ENS) and the central nervous system (CNS) and is essential for preserving homeostasis of the gastrointestinal tract, the CNS, and the gut microbial system [111,112]. This communication is made possible through the autonomic nervous system (ANS) [112,113,114] and, more specifically, by the vagus nerve (VN), which extends from the brain stem through the digestive tract and controls nearly every aspect of the transit of digested material through the intestines [112,113,114,115]. The VN is a mixed nerve made up of 80% afferent and 20% efferent fibers and is the main part of the parasympathetic nervous system [116]. The VN detects metabolites produced by the microbiota through its sensory pathways, conveying this information from the gut to the CNS. Within the CNS, this information is processed within the central autonomous network. Subsequently, the CNS generates a response, which can be appropriate or inappropriate depending on the integration of the gut-derived signals [116]. It is widely known that the VN controls the inflammatory tone of the gastrointestinal tract through a cholinergic anti-inflammatory channel. This system can reduce intestinal permeability and dampen peripheral inflammation, which likely modifies the makeup of the microbiota [116,117]. While acute exercise increases sympathetic tone, chronic exercise is well known to impact the ANS in the long-term, increasing vagal and overall parasympathetic tone [116,118,119]. Multiple rodent studies reported a clear decrease in resting heart rate, caused by an increased vagal tone, after chronic aerobic exercise [118]. Heart rate variability (HRV), which is the variation in heartbeats between one another, is a reliable measure of the ANS’s functionality and ability to respond to both internal and external stimuli [120]. As further discussed below, norepinephrine release stimulates sympathetic activation, increasing heart rate and contractility, and enhancing the heart’s responsiveness to physical and mental stress [120]. At rest, the parasympathetic nervous system dominates, releasing acetylcholine to lower the heart rate, reduce contractility, and slow conduction. Higher parasympathetic activity, which is critical for restorative processes and the primary cause of heart rate variations, is reflected in a larger HRV [120]. A higher HRV is associated with higher gut microbial diversity and specific species such as *L. incertae sedis* abundance in the healthy population [121,122]. Exercise training is well known to increase HRV in the healthy population [120,123] and could be linked to an increase in vagal tone that may promote a preferential anti-inflammatory milieu at the intestinal luminal interface [52]. Though additional research is necessary to establish this hypothesis. 

Furthermore, the hypothalamic–pituitary–adrenal (HPA) axis and the sympatho–adreno–medullary (SAM) axis work together to manage responses to physical and mental stress, like exercise [115]. The SAM system, part of the sympathetic division of the ANS, initiates the fight/flight response by releasing epinephrine from the adrenal gland, boosting heart rate, blood pressure, and energy metabolism [115]. Meanwhile, the HPA axis triggers the release of cortisol through a cascade involving the hypothalamus, pituitary gland, and adrenal glands [113,114]. Acute exercise, especially beyond 60% of maximum oxygen uptake or lasting over 90 min, activates the HPA axis, leading to increased hormone release, with intensity exacerbating this effect [124]. Multiple intervention studies in animals, healthy humans, and athletes concluded that acute, moderate, and high-intensity exercise increase levels of cortisol; however, chronic exercise at this intensity resulted in a reduction in basal cortisol concentrations compared to pre-training levels [113,124,125]. This reduction could be attributed to the increased conversion of active cortisol to inactive cortisone with regular exercise [125]. In addition to physical stress caused by acute exercise, athletes in pre-competition periods suffer from high psychological stress that also triggers HPA axis activation [115]. Similarly, an animal study showed that, compared to voluntary exercise, which reduced symptoms, moderately forced treadmill running for 40 min, five times per week increased colitis symptoms in mice, suggesting that forced exercise may have been viewed by the mice as a psychological stressor [113]. The same results were found for catecholamines, in which plasma epinephrine and norepinephrine concentrations increased acutely after exercise and decreased with regular exercise in healthy adults [124]. All these hormonal responses to exercise could impact the microbiota profile of individuals engaging in specific intensities or durations of PA [8]. Animal studies have shown the effect of stress-related hormones such as CRF, ACTH, catecholamines, and cortisol produced through the SAM and HPA axes on the gut microbiota [126]. A significant reduction in bacteria at the genus level, such as Lactobacillus and Bifidobacterium, was found under different stress conditions [126]. In line with animal evidence, Keskitalo et al. found that in infants at 2.5 months of age, saliva cortisol stress response was correlated with gut microbiota diversity but not composition [127]. Moreover, the alpha and beta diversity of the gut microbiota in humans was found to be adversely correlated with post-stressor salivary cortisol [126]. Additionally, cortisol can modify intestinal permeability, gastrointestinal transit time, and nutritional availability, all of which can have an impact on the diversity and composition of the gut microbiota [128]. Stress hormones may also influence the host epithelium, which in turn may impact the gut microbiota. It is widely established that cortisol, catecholamines, and ACTH promote bacterial adhesion to the mucosa of the gut and aid in the uptake of microbiota into Peyer’s patches [126]. These lines of evidence demonstrate how the gut microbiota is modulated by stress hormones that are produced by the SAM and HPA axes. However, more research is necessary to explore the impacts of acute and chronic exercise on stress hormone release and their influence on the gut microbiome.

#### 1.3.4. PA and Muscle Fitness 

Skeletal muscles possess metabolic and endocrine capabilities that may impact gut microbial populations, while microbes may influence skeletal muscle through various signaling pathways [129]. Metabolites from gut bacteria, such as SCFA, secondary bile acids, and neurotransmitter substrates, serve as fuel sources and inflammation regulators, affecting muscle development, growth, and maintenance [129]. The bidirectional gut–muscle axis establishes the reciprocal interactions between microorganisms, metabolites, and the muscle. A class of peptides generated from skeletal muscle called myokines may be crucial in this process [130]. Myokines, such as IL-6, IL-10, IL-1 receptor antagonist (Ra), and apelin, are produced by contracting muscle fibers and have diverse pleiotropic and local effects [130,131].

Interleukin (IL)-6, the most abundant myokine [132], depends on exercise intensity, duration, muscle mass engaged, and muscle glycogen levels [133,134]. However, IL-6 is released into the bloodstream in significant amounts during exercise [131,133]. Despite its classification as an inflammatory cytokine, muscle-derived IL-6 acts anti-inflammatory [132], promoting the secretion of other anti-inflammatory cytokines such as IL-10, IL-1Ra, and TNF-R [135]. While it is established that inflammation-related diseases alter the gut microbiota, the impact of muscle-released IL-6 and other myokines on the gut microbiome remains unexplored [8]. Nevertheless, elevated IL-6 levels can affect the gut environment by stimulating IELs to secrete GLP-1, an incretin hormone that enhances insulin secretion, decreases intestinal motility, and promotes satiety, potentially influencing nutrient availability [136]. As previously mentioned, changes in gut motility have long been recognized to impact gut microbiota composition.

Another myokine produced during exercise, apelin, controls energy metabolism in various organs and functions as a G protein-coupled receptor (APJ receptor) that affects biological processes [137]. Chao et al. demonstrated that exercise improves epithelial shape and homeostasis in mice, particularly in the villus and crypt of the duodenum, which is mediated by APJ activation [137]. However, more research is needed to determine whether myokines can impact the gut microbiota [129].

Conversely, a different myokine called myostatin (MSTN) is a key regulator of muscle growth that controls the quantity and size of muscle fibers [138]. As MSTN inhibits the formation of skeletal muscles, large increases in muscle mass and strength have been seen in animals lacking MSTN [139]. MSTN is mostly expressed in skeletal muscles [139], although it is also present in smooth muscles, such as the gut, where it is involved in a number of metabolic activities [140]. Pei et al. showed that the gut microbiota composition in MSTN mutant pigs is altered [141]. Similarly, Luo et al. discovered that deletion of the MSTN gene modifies the gut microbiome, promoting the formation of fast-twitch glycolytic muscle in mice [140]. Interestingly, resistance training for a minimum of five weeks was found to be beneficial in lowering myostatin levels in healthy people [142].

Another possible way in which muscle contractions may affect the gut microbiota is through a process known as mitochondrial crosstalk [129]. Muscle mitochondria can trigger intestinal epithelial cells and intestinal immune cells by generating reactive oxygen species (ROS) and reactive nitrogen species (RNS), thereby modifying signaling within the digestive tract [129]. Mitochondrial ROS production plays a role in regulating the gut microbiota by influencing intestinal barrier function and mucosal immune responses [143]. However, further exploration is needed to understand how exercise impacts the mitochondrial-microbiota pathway. 

Lactate, an intermediate product of energy metabolism, is considered a key stress-related molecule in human physiology [144]. Due to the fact that higher-intensity exercise accelerates glycolysis in skeletal muscles, lactate levels rise during exercise in an intensity-dependent manner [145,146]. However, lactate is also produced by the gut microbiota, especially by lactic acid-producing bacteria in the small intestine and colon [147]. During exercise, lactate in the bloodstream can penetrate the intestinal epithelium and reach the gut lumen, where lactate-metabolizing bacteria break it down. The proliferation of lactate-metabolizing bacteria in the gut may be impacted by circumstances in which lactate levels are elevated [148,149]. This interaction between lactate levels and gut microbiota is observed in various populations, including obese individuals and highly trained athletes [150]. Lactate can be metabolized by *Veillonella atypica*, the *Eubacterium hallii* group, *Anaerobutyricum hallii*, Anaerostipes, and numerous other bacterial species, producing SCFAs and other intermediates that, following an exercise period, enrich particular bacterial populations and add to microbial diversity [150]. It has also been demonstrated that lactate inhibits the growth of certain harmful bacteria, such as Escherichia coli, and that it can accumulate in large quantities in the gut of healthy newborns, indicating the predominance of bifidobacteria that produce L-lactate [147]. Nevertheless, further research is necessary to establish these mechanisms.

## 2. CKD

### 2.1. Gut Dysbiosis in CKD: The Gut–Kidney Axis

Growing evidence suggests a bidirectional relationship between disruptions in the gut microbiome and chronic diseases, including chronic kidney disease (CKD) [151,152,153]. CKD, which is defined as abnormalities in kidney structure or function present for >3 months, is a major health concern that currently affects approximately 8–16% of the population [154,155]. The incidence of CKD is rising worldwide, with CKD emerging as one of the most prominent causes of death [156].

Alterations in gut microbiota composition, diversity, functional capacity, and metabolic activity are often linked with CKD onset and progression [157,158]. On the other hand, the advancement and worsening of CKD significantly impact various physiological systems, including the gastrointestinal system [159]. It is widely established that CKD and alternations in the gut microbiome go hand in hand [157]. Gut dysbiosis, as previously defined above, manifests in three different ways, namely: (i) the depletion of beneficial organisms; (ii) the proliferation of potentially harmful organisms; and (iii) the reduction in overall microbial diversity [16]. As a result, gut dysbiosis causes pathogenic bacteria to proliferate, which raises the amounts of bacterial metabolites and breakdown products that seep into the host circulatory system and trigger persistent immunological activation [160].

Unlike healthy individuals, where protein assimilation (i.e., protein digestion, metabolism, and absorption) primarily takes place in the small intestine, patients with CKD experience impaired protein assimilation, promoting colonic protein (proteolytic) fermentation [151,161,162]. Kidney dysfunction causes the accumulation of numerous metabolites, commonly referred to as uremic retention molecules (URMs). When kidney function declines, URMs accumulate in the serum and increase the risk of cardiovascular disease and mortality [163,164]. To date, over 150 URMs have been identified and classified by The European Uremic Toxin Work Group, which classifies URMs both by their physical and chemical features or origin [165,166,167]. The latter is classified as endogenous (mammalian metabolism), exogenous (diet), or microbial [168]. Notably, gut microbial metabolism is increasingly acknowledged as a major contributor to URM production [163,169]. Several of the most harmful URMs originate from the gut microbes, including indoxyl sulfate (IS), p-cresyl sulfate (pCS), amines, and trimethylamine N-oxide (TMAO) [159,170]. There might be a bidirectional cause–effect relationship where URM accumulation alters the gut microbiota composition and function, while dysbiosis in turn leads to increased URM production [171]. More specifically, the transition towards a proteolytic fermentation pattern in CKD contributes to the rise in microbiota-derived URMs [168]. In summary, the interaction between the gut microbiota and the kidneys represents a bidirectional relationship. On one hand, the pathophysiology of CKD might contribute to the depletion of resident microbiota, while on the other hand, gut dysbiosis might influence the progression of CKD [172].

CKD-associated dysbiosis is identified by alternations in gut microbiota composition and diversity [173,174,175]. As mentioned above, a shift towards more proteolytic bacteria is seen in humans [168], with an increased abundance of species that are poorly present in healthy conditions [173,174]. In addition, a reduction in SCFA-producing bacteria and an increase in potential pathogens are detected in the CKD population [173,174,175]. Furthermore, Liu et al. showed relative abundances of *Ralstonia* and *Porphyromonas*, which were negatively correlated with the estimated glomerular filtration rate (eGFR) [174]. Liu et al. also showed that, in patients with CKD, the abundance of the genus *Akkermansia muciniphila* was significantly reduced [158]. The latter is of importance due to the fact that, as mentioned above, *Akkermansia muciniphila* has been shown to have implications for mucus thickness and intestinal barrier function. In CKD-associated dysbiosis, the intestinal barrier function is also disturbed, and with an increasing CKD stage, there is a rise in bacterial components seen in the circulation [159]. In conclusion, a tight correlation between gut dysbiosis, CKD, and CKD progression has been suggested in several studies [175].

### 2.2. PA and the Gut in CKD: A Model of Physical Inactivity 

Physical inactivity, defined as being less physically active than 150 min of moderate-intensity activity per week [176], is a major problem in patients with CKD [177]. Several observational studies in large cohorts concluded that physical inactivity was highly prevalent across all stages of CKD, and PA level worsened with CKD progression [178,179,180]. There exists a well-established link between physical inactivity and poor outcomes in individuals with CKD [180]. Reduced PA levels are not only associated with declining kidney function, more readmissions, and higher all-cause mortality in CKD, but also with gut dysbiosis in the global population [177,181]. Where PA alters the gut microbiome in a positive way, physical inactivity alters the gut microbiome towards a more detrimental gut microbiota composition and a reduction in SCFA production in healthy humans [182]. New insights into the gut microbiome and the detrimental effects of its imbalance, including the generation of gut-derived URMs, have led to the exploration of various therapeutic approaches that may be able to restore symbiotic gut conditions [52]. Whether PA interventions could be effective in counteracting gut dysbiosis and the production of gut-derived URMs in CKD still needs to be further explored. Unfortunately, the limited number of previous studies only evaluated the effects of exercise on URMs and lacked analyses of gut microbiome alternations. Initially, no studies have investigated the impact of PA on the gut microbiome in CKD patients. However, certain studies have explored the potential of PA in lowering URMs. De Brito et al. conducted a randomized controlled trial, concluding that neither aerobic exercise three times a week for three months nor resistance training three times a week during six months reduced levels of IS, p-CS, and indol-3-acetic acid in 20 and 26 HD patients, respectively [183]. Fortunately, one study is currently being performed to evaluate the effects of PA on the gut microbiota and URMs in stage 3–4 CKD patients [184].

### 2.3. Mechanisms via Which PA Could Restore Gut Dysbiosis in CKD 

**Figure 2:**
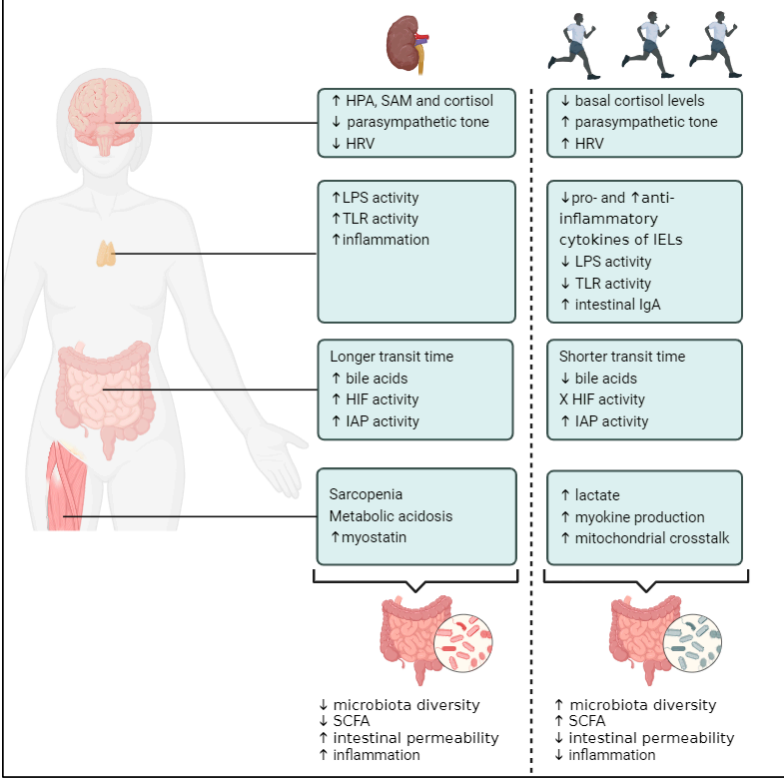
Effects of CKD on the gut microbiome and the possible therapeutic effect of chronic exercise. HPA: hypothalamic–pituitary–adrenal axis; SAM: sympatho–adreno–medullary; HRV: heart rate variability; IEL: intraepithelial lymphocytes; LPS: lipopolysaccharide; TLR: toll-like receptors; IgA: immunoglobulin A; HIF: hypoxia inducible factor; IAP: intestinal alkaline phosphatase; SCFA: short-chain fatty acids.

All four different levels (as discussed in Section 1.3) are altered in CKD and contribute to gut dysbiosis. The alternations at these four different levels seen in CKD are discussed below. Furthermore, the possible therapeutic potential of PA is hypothesized, as visualized in Figure 2. 

#### 2.3.1. CKD, PA and Gastrointestinal Fitness

Patients with CKD, and particularly hemodialysis (HD) patients, often suffer from a slow colonic transit time and frequent constipation [185,186]. Reduced dietary fiber intake, water restriction, lack of PA, medication usage (i.e., phosphate binders), and decreased gastrointestinal motility might all contribute to the higher incidence of constipation in CKD [186,187]. As previously noted, a growing body of evidence suggests that intestinal transit time plays a crucial role in influencing the composition and function of the gut microbiota [45]. Slower intestinal transit time can lead to an imbalance between glycolytic and proteolytic bacteria, ultimately shifting microbial metabolism from glycolysis to protein fermentation patterns [174]. A recent systematic review and meta-analysis reinforced that exercise improves constipation in the general population [188]. However, whether exercise relieves constipation or increases colonic transit time in patients with CKD is still unclear [185,189]. 

In normal conditions, kidney clearance accounts for two-thirds of total body clearance of uric acid, whereas intestinal excretion accounts for approximately one-third of total body clearance of uric acid [190]. Progressive kidney failure results in higher concentrations of urea in the blood, resulting in an increased urea influx into the gut lumen [191]. Subsequently, urea is converted to ammonia via bacterial urease, which are byproducts of bacteria that metabolize urea to ammonia and carbon dioxide [71]. The accumulation of ammonium in the gut raises the intestinal pH and weakens the junctions of intestinal cells, leading to increased gut barrier permeability (further discussed below) [192]. Production of fermentation products, such as SCFA, and their distribution depend on intestinal pH [193]. However, due to the fact that exercise-induced lactic acidosis can worsen intestinal pH, this topic is further discussed at the level of the muscle.

Multiple animal and clinical studies have shown that CKD is associated with altered bile acid balance, including elevated serum bile acid levels [194,195]. This is significant since bile acids independently increase the likelihood of poor renal outcomes in people with CKD [196,197]. Both the variable expression of bile acid transporters and the increased bile acid synthesis are potential processes contributing to raised bile acid levels in CKD patients [194,198]. Nevertheless, one important contributor to the elevated bile acid levels in patients with CKD is the decreased filtration of bile acids through the kidneys [198]. However, further investigation of the underlying mechanisms is necessary [194,198]. Feng et al. showed by means of metagenomic sequencing that the marked decline in gut microbiome diversity and richness of CKD rats goes along with dysregulation and altered enzymatic activities of bile acids [199]. Wu et al. observed an increased abundance of genes associated with the conversion of primary to secondary bile acids in the gut microbiome of CKD patients [200]. With elevated bile acid levels being a risk factor and bile acid metabolism being a modulator of gut microbiome composition, the depletion of bile acids in the CKD population is of importance. In the general population, as mentioned above, it is well known that exercise has positive effects on bile acid levels, but current studies in CKD patients are lacking. Therefore, further research on the role of exercise on bile acid metabolism in the CKD population and the accompanied effects on gut dysbiosis is needed.

The expression of genes that are responsible for adaptation to hypoxia is controlled by HIF [71]. From a physiological standpoint, hypoxia and the activation of HIF responses play a significant role in regulating the metabolism of intestinal epithelial cells, as well as in the regulation of intestinal epithelial barrier function and gut microbiota segregation in humans and mice [70]. However, prolonged activation of HIF results in intestinal injury and inflammation [71,201,202,203]. On the other hand, intestinal inflammation causes hypoxia, consequently activating HIF [204]. It is widely established that intestinal inflammation is highly common in CKD [205]. This increased inflammation stems from various factors. Firstly, there is an accumulation of gut-derived URMs, which promote inflammatory reactions and leukocyte stimulation in stage 3–4 CKD patients [205]. Secondly, the influx of urea and the production of bacterial urease lead to the breakdown of epithelial junctions in the colon, sparking localized inflammation through cytokine production [191]. Lastly, the reduced carbohydrate fermentation results in the deprivation of anti-inflammatory SCFAs in CKD [190]. Besides this intestinal inflammation, the increased amount of ammonia in CKD patients’ intestinal lumen leads to cellular stresses and directly interacts with HIF [204], in which HIF-1α forms a negative feedback loop to counteract ammonia toxicity [71]. Studies clearly demonstrating that intestinal HIF activity is elevated in CKD are currently missing, and the acute and chronic effects of exercise on intestinal HIF activity are not yet investigated in the CKD population.

Research on IAP in CKD is presently limited [206], which is notable considering that IAP, an endogenous protein expressed by the intestinal epithelium, is thought to be crucial for homeostasis in the gut [207]. Contrary to expectations, some studies reported increased IAP activity in patients with CKD [206,208,209]. The mechanisms underlying this rise in IAP remain elusive; however, it could potentially stem from the liver’s incapacity to clear this circulating enzyme, particularly in CKD accompanied by liver disease [206]. Furthermore, the kidneys could also be responsible for total serum alkaline phosphatase elevations in some patients [206]. However, further research is required to gain insights into IAP levels and functions in the uremic gut of CKD patients and the possible effects of PA. 

#### 2.3.2. CKD, PA and Fitness of the Immune System 

The maintenance of gut homeostasis relies on the strategic positioning of the GALT at the mucosal interface, which facilitates surveillance and the innate and adaptive immune functions of immunocytes [190]. In CKD, the activity of the GALT is disrupted [210] with recent research revealing significant alterations in the composition, structure, and function of the intestinal lymphatic system of CKD rodents, including heightened lymph flow, lymph angiogenesis, and the transport of lipoproteins and pro-inflammatory mediators [210].

The above-mentioned changes could, among others, possibly manifest due to the impaired intestinal barrier in CKD patients, influencing both mucosal inflammation and epithelium integrity [175]. The mechanisms by which CKD impairs the intestinal barrier are multifactorial and not yet completely understood; however, several mechanisms take place. A review by Meijers et al. clearly described the relationship between CKD and intestinal permeability regarding several rodent studies [190]. Vaziri et al. observed significant changes in the structural components of the intestinal barrier of rodents with CKD, particularly intense lymphocyte infiltration into the lamina propria and loss of tight junction proteins [211,212,213]. Georgopoulou et al. observed a reduction in the intestinal expression of occluding and claudin-1, key molecular components of tight junctions. This suggests a possible cellular mechanism underlying intestinal barrier dysfunction in CKD patients [214]. A compromised intestinal barrier results in a heightened translocation of bacterial metabolites and bacterial degradation products across the intestinal barrier into the system’s circulation [190]. McIntyre et al. demonstrated that circulating LPS levels are heightened in all stages of CKD, reaching their maximum in dialysis patients [215]. Szeto et al. demonstrated a link between circulating LPS and systemic inflammation as well as indicators of atherosclerosis in PD patients [216]. Adda-Rezig et al. recently found disparities in the capacity to neutralize circulating LPS and its subtypes between end-stage kidney disease (ESKD) patients and healthy controls [217]. The latter showed a higher activity of LPS in ESKD patients compared to controls, attesting to better LPS neutralization in the latter, which was explained by both lower concentrations of high-density lipoprotein (HDL) and higher phospholipid transfer protein (PLTP) activity in ESKD patients. HDL mainly helps remove LPS from the bloodstream by binding to it, which stops LPS from activating monocytes and macrophages through TLR4. Additionally, HDL can help release LPS already bound to myeloid cells, further reducing its activity. Similarly, PLTP boosts the binding of LPS to lipoproteins, which decreases its ability to cause inflammation [218]. In CKD, the presence of pro-inflammatory LPS in the bloodstream might increase PLTP activity. This heightened activity aims to transport LPS to HDL for neutralization. However, the reduced concentration of HDL limits its ability to effectively neutralize LPS, leading to ongoing inflammation [217]. Adda-Rezig et al. noted that impaired LPS clearance exacerbates LPS-induced inflammation in ESKD, leading to elevated levels of inflammation biomarkers and heightened TLR4-mediated cytokine release by monocytes [217]. Conversely, Ando et al. observed a significant decrease in TLR4 expression in CKD patients, irrespective of predisposition to previous bacterial infection [219]. However, they found impaired LPS-induced cytokine synthesis in CKD patients predisposed to bacterial infections, with each cytokine response showing a significant correlation with TLR4 expression in monocytes [219]. Koc et al. reported lower percentages of monocytes expressing TLR4 in CKD patients compared to controls [220]. In summary, reduced TLR4 expression in CKD patients may compromise their response to bacterial infections, regardless of their prior infection history. However, Koc et al. also noted higher intensities of TLR2 on CD14+ monocytes in CKD patients compared to controls, suggesting potential chronic low-grade monocyte activation in these patients [220]. LPS-induced monocyte/macrophage activation and systemic inflammation play a pivotal role in Gram-negative sepsis, and this phenomenon could elucidate the persistent systemic inflammation observed in CKD [221]. As previously mentioned, chronic exercise could be linked to enhanced intestinal barrier integrity and reduced LPS and TLR activity, thereby influencing the gut microbiome. Unfortunately, there is currently no available data on the effects of exercise on gut barrier integrity, LPS activity, or TLR activity in CKD patients. Nevertheless, it is well established that exercise interventions exert significant anti-inflammatory effects across the spectrum of CKD [222].

Regarding IgA, IgA nephropathy (IgAN) stands as the most prevalent primary glomerulonephritis globally [223]. Given that IgA is primarily synthesized by mucosa-associated lymphoid tissue and is abundant in the intestine, IgAN has been linked to gut dysbiosis, intestinal barrier disruption, and bacterial translocation into the bloodstream [224]. However, due to its complexity, IgAN is out of the scope of this review. 

#### 2.3.3. CKD, PA and Fitness of the Nervous System 

The vagus nerve (VN) serves a pivotal role in the communication between the gut and the central nervous system (CNS) [112,113,114]. It regulates gastrointestinal inflammation through a cholinergic anti-inflammatory pathway (CAP) and likely influences microbiota composition [116,117]. Increasing vagal tone through exercise may create a more anti-inflammatory environment in the intestinal lining. Consequently, exercise could lead to reduced intestinal permeability, preserving gut mucosa, and decreasing endotoxin translocation in the healthy population [21,116,118]. Autonomic dysfunction, marked by an imbalance between sympathetic and parasympathetic nerve activity, is frequently observed in individuals with CKD and is associated with a bad prognosis [225,226,227]. In CKD, increased sympathetic activity is observed and is associated with a reduced parasympathetic tone [228]. The latter may have a significant clinical impact, including delayed gastric emptying, intestinal dysfunction, and immune system dysregulation [228]. Recent discoveries have unveiled a link between HRV and the onset of renal impairment, suggesting an association between autonomic dysfunction and CKD [229]. In a longitudinal study conducted by Brotman et al., they identified a significant association between low HRV and the development of renal impairment. This association remained significant even after adjusting for other factors known to contribute to renal failure, such as diabetes, hypertension, lipid profiles, baseline kidney function, and parameters associated with insulin resistance and obesity [229]. Regarding the effects of exercise, Jeong et al. were the pioneers in demonstrating that in older, sedentary persons with mild to severe CKD, exercise training slows the course of resting sympathetic nervous system overactivity [230]. A review by Barcellos et al. revealed that multiple studies in both pre-dialysis and HD patients found significant improvements in HRV after exercise interventions [231]. A Cochrane meta-analysis similarly revealed a significant improvement in HRV index following 6 months of mixed aerobic and resistance training in HD patients [232]. This is of major importance due to the fact that, as mentioned before, HRV is linked to increased gut microbial diversity [121]. However, the effect of increased HRV in CKD patients and the possible effect of reduced HRV by exercise on the gut microbiome are not yet explored. 

Various changes in the function of the HPA axis have been documented in individuals with CKD [233], leading to increased cortisol secretion and local amplification of its effects in tissues [234]. These changes in cortisol levels are caused by a decrease in renal filtration of cortisol metabolites, a decrease in renal enzymatic inactivation, insufficient negative feedback mechanisms, and dysregulation of the HPA axis [233]. Cortisol metabolism and the excretion of cortisol byproducts through urine may be further hindered if renal function continues to deteriorate [234]. Despite the modest increase in cortisol levels, sustained mild cortisol excess is increasingly acknowledged as a risk factor for morbidity and mortality in CKD [233]. Consequently, it is important to implement therapies that lower cortisol levels in this population. Exercise is one such intervention because long-term exercise increases the inactivation of cortisol into the inert steroid cortisone [125]. Unfortunately, studies evaluating the effectiveness of exercise as a therapy to reduce cortisol and the accompanied effects on the gut microbiome are currently lacking in the CKD population.

#### 2.3.4. CKD, PA, and Muscle Fitness

Sarcopenia, characterized by the loss of muscle mass, quality, and function, is widespread among individuals with CKD and associated with heightened mortality and morbidity, frailty, and hospitalizations [235,236,237]. CKD-associated sarcopenia is multifactorial and caused by several mechanisms, amongst others, inflammation, oxidative stress, insulin resistance, metabolic acidosis, physical inactivity, etc. [237,238]. Overall, individuals with acute, subacute, or chronic muscle wasting conditions exhibit a distinct gut microbial composition compared to their healthy counterparts [239,240]. This altered microbial composition is also linked to changes in metabolomics and alterations in the host gut barrier [240].

Muscle wasting results from an imbalance between skeletal muscle breakdown and synthesis, crucial for maintaining muscle homeostasis—a complex process [237], described elsewhere [241]. Metabolic acidosis, nearly universal in advanced CKD patients, exacerbates protein degradation by activating the ubiquitin–proteasome pathway, possibly hindering amino acid transport and utilization [241,242]. In healthy individuals, kidney function regulates acid–base balance through bicarbonate reabsorption and generation. As kidney function deteriorates, metabolic acidosis arises due to reduced acid-excretory capacity and high daily endogenous and exogenous acid loads [241]. The severity of acidosis typically corresponds with the extent of kidney failure and arises from decreased ammonia excretion and titratable acid elimination, along with reduced bicarbonate reabsorption and synthesis [243]. As noted earlier, metabolic acidosis is a recognized contributor to sarcopenia. This is due to the fact that it causes negative nitrogen and total body protein balances, inhibits protein synthesis, and stimulates protein breakdown [243,244]. Emerging evidence suggests that metabolic acidosis may provoke chronic inflammation [244]. A single session of resistance exercise was found to enhance protein synthesis in the muscles of HD patients [245], while long-term combined strength and endurance training slowed muscle catabolism in dialysis patients [246]. However, there is speculation that exercise-induced lactic acid production in non-dialyzed CKD patients could worsen metabolic acidosis, potentially offsetting the benefits of exercise [247].

It is evident that decreased skeletal muscle mass relates to a decrease in myokine secretion. Numerous studies have demonstrated that sarcopenia leads to lower levels of myokines released from skeletal muscle fibers, subsequently promoting an increase in pro-inflammatory cytokines and impaired glucose and lipid metabolism [248]. CKD is linked with dysregulated myokine activity and a systemic rise in cytokines [249]. Regarding the myokine Irisin, both animal and human studies revealed a link with CKD. Kawao et al. demonstrated that kidney failure in mice is associated with a reduction in the expression of irisin in the gastrocnemius muscles [250]. A recent review discussed the role of irisin in diabetic nephropathy, with several human studies showing reduced levels of irisin in patients with diabetic nephropathy [251]. Wang et al. demonstrated significantly reduced serum irisin levels in diabetic patients with microalbuminuria and macroalbuminuria compared to those with normal albuminuria, which decreased further with increasing proteinuria and declining GFR [251]. Conversely, Mageswari et al. demonstrated an increase in circulating irisin levels in diabetic nephropathy (DN) patients compared to diabetics without nephropathy, suggesting a potential role for irisin as an indicator of DN progression [252]. In conclusion, the current findings, according to the myokine Irisin, are contradictory, and further research is necessary. Importantly, myostatin, a myokine that suppresses the growth of skeletal muscles, plays a crucial role in the loss of muscle mass in CKD [139]. CKD is associated with increased expression of myostatin [139]. This is significant because lower myostatin levels have a beneficial effect on the gut microbiota [140,141]. Therefore, it can be stated that an increase in this myokine negatively influences the gut microbiome. However, evidence is currently lacking. Fortunately, an extreme model of resistance exercise (muscle overloading) in rats with CKD documented a downstream effect of myostatin, while 18 weeks of endurance exercise further reduced myostatin [139].

Finally, there are other changes in metabolomics observed in muscle wasting, including shifts in quorum sensing molecules (QSMs) [240]. QSMs are bacterial products produced by living bacteria and exhibit increased production under “stress” conditions. Aside from their role in intra-bacterial communication, some QSMs have been shown to traverse the gut barrier and act as potential signals in bacterial–host communication [240]. For instance, Spiegeleer et al. demonstrated the effects of specific QSMs on muscle cells, suggesting that QSMs may be involved in the gut–muscle axis and could contribute to muscle wasting diseases [240]. However, this topic falls beyond the scope of this review.

## 3. Conclusions

In conclusion, disturbances in the gut microbiome are widely associated with the development and progression of CKD. Physical inactivity, which is highly prevalent among patients with CKD, is an important modulator of the gut microbiome and may contribute to gut dysbiosis in the CKD population. PA can potentially mitigate gut dysbiosis in CKD through mechanisms involving the gut, the immune system, the autonomic nervous system, and muscles. However, evidence is currently lacking, and additional research is required to substantiate this hypothesis and fully understand the impact of physical (in)activity on gut health in the CKD population.

## Data Availability

Not applicable.
